# Inter-individual, hemispheric and sex variability of brain activations during numerosity processing

**DOI:** 10.1007/s00429-023-02747-3

**Published:** 2024-01-10

**Authors:** Zhongyao Zang, Xiaoyue Chi, Mengkai Luan, Siyuan Hu, Ke Zhou, Jia Liu

**Affiliations:** 1 Beijing Key Laboratory of Applied Experimental Psychology, National Demonstration Center for Experimental Psychology Education (Beijing Normal University), Faculty of Psychology, Beijing Normal University, Beijing, 100875 China; 2 Department of Psychology, Shanghai University of Sport, 650 Qing Yuan Huan Road, Shanghai, 200438 People’s Republic of China; 3 Tsinghua Laboratory of Brain and Intelligence, Department of Psychology, Tsinghua University, Beijing, 100084 China

**Keywords:** Numerosity, Inter-individual difference, Hemispheric difference, Sex difference, Functional probabilistic atlas

## Abstract

Numerosity perception is a fundamental and innate cognitive function shared by both humans and many animal species. Previous research has primarily focused on exploring the spatial and functional consistency of neural activations that were associated with the processing of numerosity information. However, the inter-individual variability of brain activations of numerosity perception remains unclear. In the present study, with a large-sample functional magnetic resonance imaging (fMRI) dataset (*n* = 460), we aimed to localize the functional regions related to numerosity perceptions and explore the inter-individual, hemispheric, and sex differences within these brain regions. Fifteen subject-specific activated regions, including the anterior intraparietal sulcus (aIPS), posterior intraparietal sulcus (pIPS), insula, inferior frontal gyrus (IFG), inferior temporal gyrus (ITG), premotor area (PM), middle occipital gyrus (MOG) and anterior cingulate cortex (ACC), were delineated in each individual and then used to create a functional probabilistic atlas to quantify individual variability in brain activations of numerosity processing. Though the activation percentages of most regions were higher than 60%, the intersections of most regions across individuals were considerably lower, falling below 50%, indicating substantial variations in brain activations related to numerosity processing among individuals. Furthermore, significant hemispheric and sex differences in activation location, extent, and magnitude were also found in these regions. Most activated regions in the right hemisphere had larger activation volumes and activation magnitudes, and were located more lateral and anterior than their counterparts in the left hemisphere. In addition, in most of these regions, males displayed stronger activations than females. Our findings demonstrate large inter-individual, hemispheric, and sex differences in brain activations related to numerosity processing, and our probabilistic atlas can serve as a robust functional and spatial reference for mapping the numerosity-related neural networks.

**Supplementary Information:**

The online version contains supplementary material available at 10.1007/s00429-023-02747-3.

## Introduction

Estimating the number of objects in a set, namely numerosity perception, is crucial in our daily lives (Burr and Ross 2008; Piazza 2010; Dehaene et al. 1999; Feigenson et al. 2004; Piazza and Izard 2009). For example, by estimating and comparing the numbers of items in different groups, we can make more beneficial decisions, e.g., choosing a shorter queue to wait in. Over the last two decades, numerosity perception has been extensively studied, and accumulating evidence suggests that it may be innate (Xu and Spelke 2000; Berger 2011; Evans and Gold 2020; Decarli et al. 2022) and shared by both humans and many animal species (Nieder and Miller 2004; Pisa and Agrillo 2009; Piffer et al. 2012; Pica et al. 2004), and is critical for the survival and reproduction of animals and for constructing abstract and complex mathematical concepts in humans (for reviews see Piazza and Izard 2009; Burr et al. 2018).

A central topic in numerosity perception is understanding the neural mechanisms that underlie the representation and processing of numerical information. In the past two decades, significant progress has been made in applying electrophysiological and fMRI approaches to characterize the neural substrates of numerosity perception. Studies has showed that a wide range of brain structures, including the intraparietal sulcus (IPS), superior parietal lobule (SPL), insula, dorsal lateral prefrontal cortex (DLPFC), inferior frontal gyrus (IFG), inferior temporal gyrus (ITG), premotor area (PM), middle occipital gyrus (MOG) and anterior cingulate cortex (ACC), were related to numerosity processing. Among these regions, a specialized parietal-frontal network, including the IPS and DLPFC was found to be consistently activated during numerosity processing (Dehaene et al. 1999, 2003; Eger et al. 2003; Pinel et al. 2004; Hayashi et al. 2013; Pinheiro-Chagas et al. 2018; Ustun et al. 2021). Activations of other brain regions, such as the SPL (Otsuka et al. 2008; Shomstein 2012), insula (Menon and Uddin 2010; Ustun et al. 2021), ACC (Menon and Uddin 2010; Ustun et al. 2021), IFG (Hampshire et al. 2009; Hayashi et al. 2013; Zhang and Iwaki 2019), MOG (Li et al. 2019; Gandini et al. 2008), and ITG (Pinheiro-Chagas et al. 2018), was also reported in relation to the attentional, and cognitive control processes required for the numerosity task. For instance, SPL has been identified as one of the key regions for arithmetic calculations (Pinheiro-Chagas et al. 2018) and the MOG is involved in attention orientation and strategy selection during numerosity estimation (Gandini et al. 2008). The insula is linked to the difficulty level in numerosity comparisons (Ustun et al. 2021), and IFG is responsible for the joint coding of numerosity and time during decision-making (Hayashi et al. 2013). The ITG plays a crucial role in digit recognition and the early identification of problem difficulty during numerosity estimation (Pinheiro-Chagas et al. 2018), and the ACC is involved in executive control, working memory, and attention processes during numerosity perception (Ustun et al. 2021). Recently, several studies also revealed the involvement of the PM in estimating the numerosity of self-generated actions, and that the characteristics of this function closely resemble those associated with the estimation of sensory numerosity, suggesting a close link between the number and action (Kansaku et al. 2006; Nieder 2017). In short, a wide range of brain structures forms the neural basis of numerosity perception, which supported the emerging network view of the neural representation of numerosity (Arsalidou et al. 2018; Zhang et al. 2023). However, previous studies have primarily focused on identifying common patterns of brain activation across individuals, i.e., the spatial and functional consistency of neural activations, the inter-individual variability in brain activations associated with the numerosity processing remains unclear.

Several fMRI studies have attempted to address the inter-individual differences in numerosity processing. They often adopted the extreme-group approach that involves comparing the brain activations of a small experimental group of individuals with a specific condition (such as dyscalculia) to a control group of healthy individuals (Ustun et al. 2021; Gandini et al. 2008). For instance, one seminal study found that people with dyscalculia exhibited much stronger activation in the right ACC than the healthy controls in a numerosity task (Ustun et al. 2021), which suggested the possible link between ACC and numerosity perception. However, certain limitations still persist in these studies. First, the small sample size of these studies often makes it challenging to identify the relationship between brain regions and numerosity processing, and to generalize their findings to explain the great variations in numerosity processing among individuals. Another limitation is that these studies often restricted their analysis to the relationship between neural activity in the specific brain region and behavioral performance (Ustun et al. 2021; Gandini et al. 2008; Haist et al. 2015). However, little is known about the inter-individual variations in numerosity processing across the entire brain. Therefore, a comprehensive and quantitative description of inter-individual variability in brain activations (particularly at the regional level) during numerosity perception is still lacking.

Moreover, previous fMRI research has found evidence for the hemispheric asymmetry in brain activations during numerosity processing (Piazza et al. 2006; Hayashi et al. 2013; Leibovich et al. 2015). Brain activations of non-symbolic number estimation appears to be right lateralized (Piazza et al. 2006; Leibovich et al. 2015), especially in the right fronto-parietal cortical network (Leibovich et al. 2015). In other regions, the right hemisphere may also play a more prominent role in numerosity processing. For example, Hayashi et al. (2013) found that the right IFG was involved in the joint coding of numerosity and time during decision-making, while the left IFG was not. However, there is still a lack of systematic investigation into the functional asymmetry of numerosity processing.

In the present study, we aimed to quantify the inter-individual variability of brain activations during the processing of numerosity information using a classical numerosity comparison task (Suarez-Pellicioni et al. 2019) and a large sample dataset of 460 participants. First, we delineated the subject-specific activated regions of interest (ROIs) responsible for numerosity processing in each individual, including the anterior and posterior IPS, IFG, ITG, PM, MOG, ACC, and insula. Then, we created a functional probabilistic atlas to quantify the spatial variability of numerosity-related brain activities, which contained precise stereotaxic information on both inter-hemispheric and inter-individual differences. Specifically, we characterized the functional and spatial variabilities in brain activities using three features: peak location, volume size, and activation magnitude of the activated regions. Besides the inter-individual variability, the inter-hemispheric and sex differences of these features were also examined. Finally, a series of evaluations were carried out to test the reliability and robustness of our functional atlas.

## Materials and methods

### Participants

460 healthy adults (206 males) from Beijing Normal University participated in this study, who met these following inclusion criteria: 1) normal or corrected-to-normal vision; 2) no current or past psychiatric or neurologic disorders; 3) no discomfort in a confined space; 4) no metallic objects in their body. The mean age of participants was 25.89 years (standard deviation [SD] = 1.04 years; ranging from 17 to 32 years). Four subjects were excluded from further analysis due to scanner malfunction or excessive movement inside the scanner.

### Numerosity localizer task

All stimuli were generated and presented using MATLAB (MathWorks, Inc., Natick, MA) and PsychToolbox 2.54 (Brainard 1997; Pelli 1997). The stimuli were displayed on a 70 × 39.4 cm screen inside the MRI bore (resolution: 1920 × 1080 pixels, refresh rate: 60 Hz), which was positioned 120 cm away from the participant’s eyes.

**Fig. 1:**
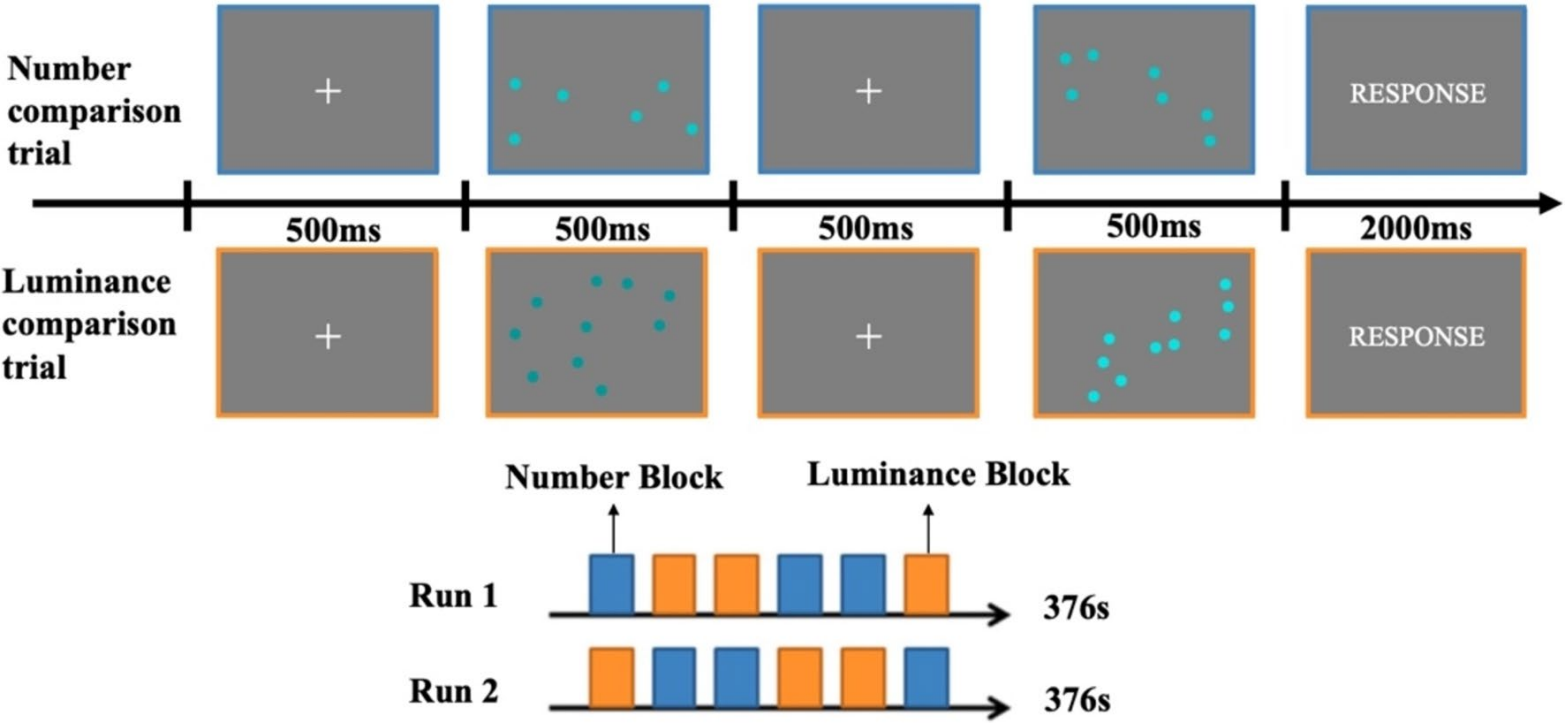
Illustration of the stimuli and procedure of the numerosity localizer task

Since existing research has revealed a considerable overlap in the neural substrates associated with both luminance and numerosity perception (Pinel et al. 2004; Kadosh et al. 2008; Robert et al. 2023), it is crucial to isolate the neural substrates specific to numerosity perception. To achieve this, we designed a localizer task that consisted of two conditions: number comparison and luminance comparison, as shown in Fig. 1. Employing the luminance comparison task as a control condition allowed for a more isolated examination of the brain activities specifically associated with numerosity perception. In the number comparison condition, each trial started with a 500-ms fixation point (0.34 × 0.34°) at the center of the screen, followed by the first dot array (14 × 10.4°) containing cyan dots (0.17 × 0.17°), which was presented for another 500 ms. After that, another fixation point was presented for 500 ms, followed by the second dot array presenting for another 500 ms. Participants were instructed to rapidly judge which dot array contained more dots and press the corresponding button within 2000 ms. To modulate the difficulty level in the number comparison task, we employed dot ratios of 1:2, 3:4, 5:6, 7:8, and 9:10 between the two dot arrays. The number of dots in each array was chosen from a range of 6 to 12, and all dots within both arrays exhibited uniform lightness. Therefore, the individual dot array contained either 6, 7, 8, 9, 10, or 12 dots. In this task, dot arrays were pseudo-randomly generated to ensure that subjects cannot complete the task based on the convex hull or density information (See Supplementary materials for more details; Figure S1). In the luminance comparison condition, the stimuli and procedure were the same as those in the number comparison condition, except for the following changes. Two dot arrays both contained 10 dots. The lightness of the dots in each dot array varied between (67, 151, 152) and (105, 227, 228). Participants were asked to compared which dot array had a higher luminance by pressing corresponding button. Each trial lasted 4 s. The duration of the inter-trial intervals was set at 500 ms.

The fMRI study used a blocked design and contained two runs, each lasting 376 s. Each run consisted of 6 experimental blocks, with 3 blocks for number comparison condition and 3 blocks for luminance comparison condition (Fig. 1). Each block started with a 4-s instruction display informing the participant about the condition they would perform, followed by 10 experimental trials. In addition, seven 16-s fixation blocks were interleaved with the experiential blocks. The participants were instructed to complete the task as quickly and accurately as possible. Their behavioral responses and reaction times (RTs) were recorded during the fMRI scan.

### fMRI data acquisition

Functional and anatomical MRI images were acquired at the Imaging Center for Brain Research, using a Siemens Trio 3 T whole-body scanner equipped with a 12-channel phased-array head coil. Functional images were obtained using a gradient-echo planar sequence (repetition time (TR) = 2000 ms, echo time (TE) = 30 ms, flip angle = 90°, in-plane resolution = 3.5 × 3.5 mm, FOV = 232 × 232, 30 continuous axial slices, slice thickness = 4.8 mm). Structural images were acquired using a 3D T1-weighted magnetized rapid acquisition gradient echo sequence (TR = 2530 ms, TE = 3.39 ms, inversion time = 1100 ms, flip angle = 7°). Foam pillows and extendable padded head restraints were used to limit head movement. Ear plugs were used to reduce scanner noise.

### Image preprocessing

Functional data were processed and analyzed using the fMRI Expert Analysis Tool (FEAT) available in the Oxford Center for Functional MRI of the Brain Software Library (FSL) (Smith et al. 2004). Preprocessing consisted of motion correction, Gaussian spatial smoothing with a 6 mm full width at half maximum and high-pass temporal filtering with a 120-s cutoff. To mitigate the effects of motion artifacts, participants whose functional data exhibiting translational or rotational deviations greater than 3 mm or 3°, respectively, were excluded from further analysis. In the first-level analysis, functional data were subjected to a General Linear Model (GLM) regression, incorporating not only the time series of task conditions—namely luminance and numerosity—but also the estimated motion parameters as nuisance regressors. The design matrix for these conditions was generated by convolving a canonical hemodynamic response function (HRF), modeled as a gamma function, with a boxcar function representing the timing and duration of the respective experimental conditions. In second-level analysis, data from each participant's two runs were integrated via fixed-effects analysis. The functional images were first aligned to individual structural scans, normalized to the MNI standard template, and resampled at a 2 × 2 × 2 mm resolution. We conducted contrasts between numerosity and luminance comparisons to isolate numerosity-related neural activities. Each participant's resultant statistical map, represented as a z-score, was used to delineate regions of brain activation specific to numerosity perception.

### Labeling the functional areas of numerosity

**Fig. 2:**
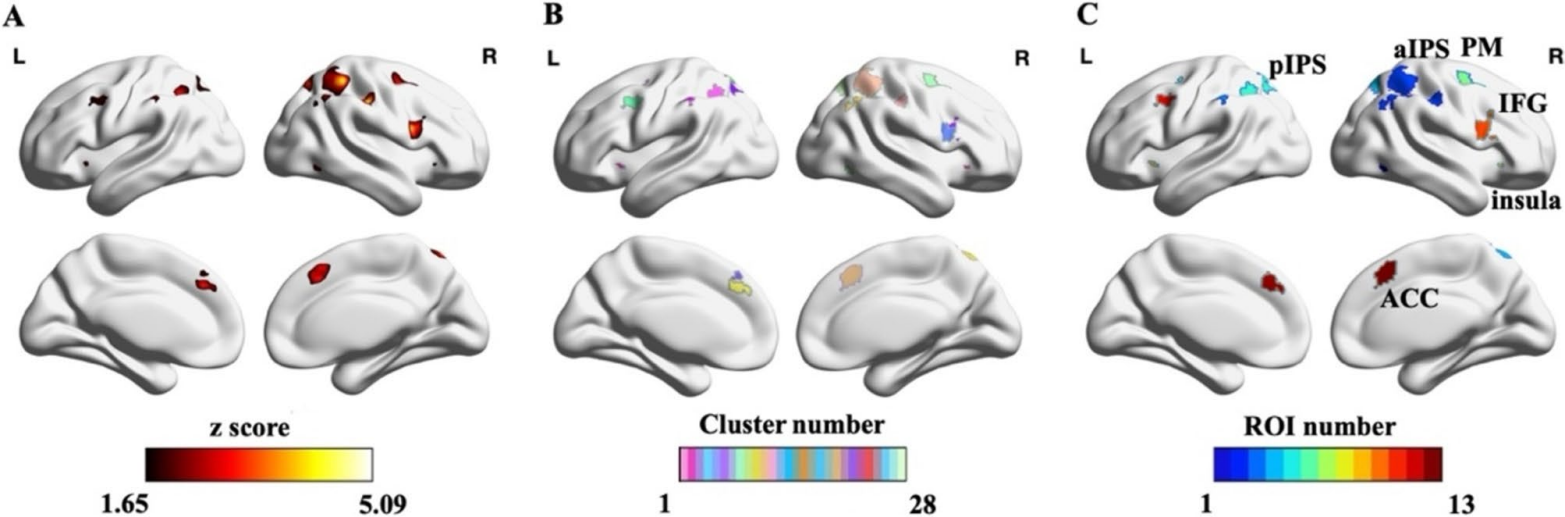
Semi-automated process used to delineate subject-specific activation regions (in one exemplar participant). **A** The individual activation map was derived by contrasting numerosity comparison condition vs. luminance comparison condition with z > 1.65 (*p* < 0.01, right-tailed, uncorrected). **B** The watershed algorithm was applied to divide individual activation map into small parcels and marked them with different colors. **C** Subject-specific ROIs were labeled based on the group-level functional reference map and anatomical landmarks

We identified eight numerosity-related regions of interest (ROIs) in both hemispheres of each participant, including the ITG, aIPS, pIPS, PM, IFG, insula, MOG, and ACC, using a semi-automated process described below (Fig. 2).

First, the activation map of each subject was thresholded at z > 1.65 (*p* < 0.01, right-tailed, uncorrected) (Fig. 2A). Then, we employed the Watershed algorithm to partition the activation map into small parcels (Meyer 1994) to avoid subjective determination of the boundaries between ROIs (Fig. 2B). Finally, subject-specific ROIs were selected within these parcels by two raters manually (one main rater and one assistant rater) based on the group-level functional reference map and the MNI152 template (Fig. 2C). Results of main rater were used for further analysis, while the assistant rater’s results were used to evaluate the inter-rater stability of these ROIs.

**Fig. 3:**
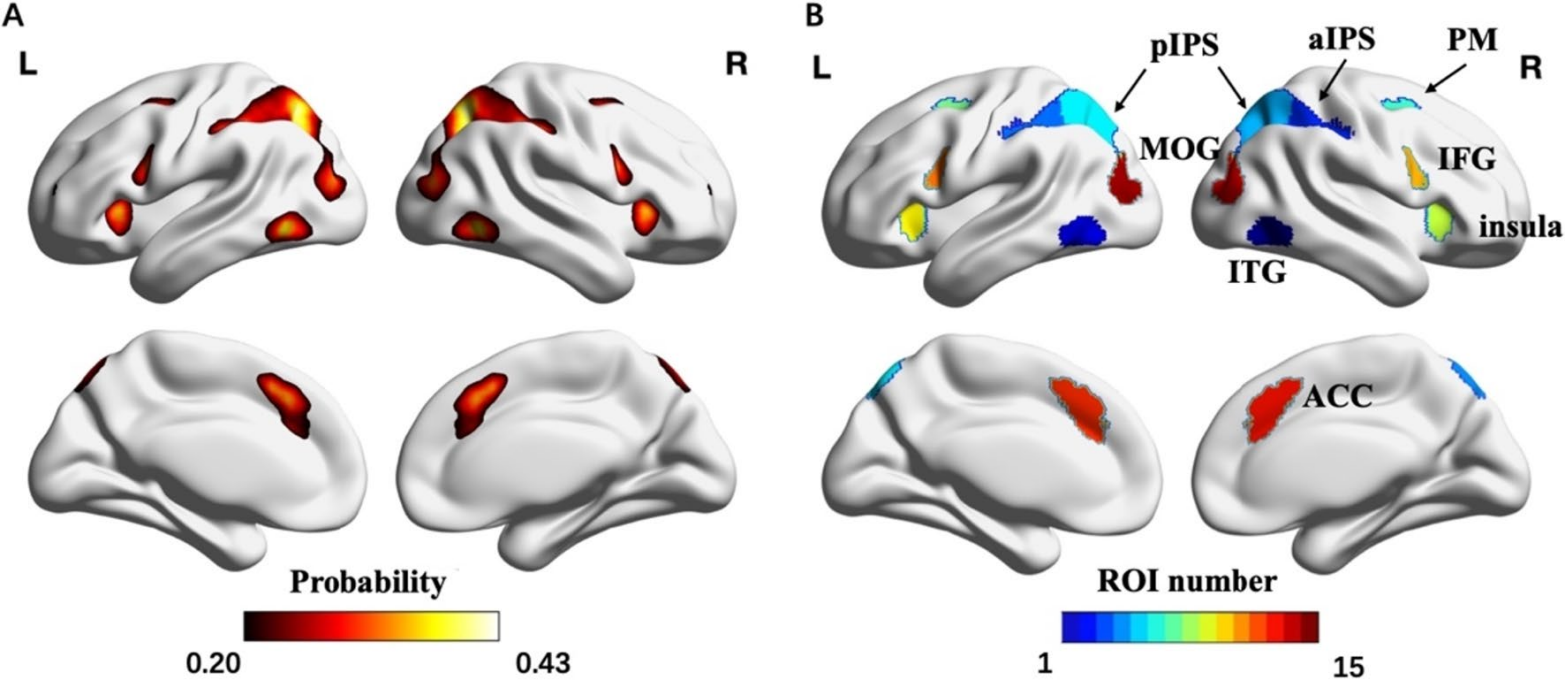
Procedure used to create the group-level functional reference map. **A** The symmetric probabilistic map. We first averaged the binary activation map (threshold: z > 1.65) of all participants to obtain the averaged probabilistic map. Then, we flipped the averaged probabilistic map, added it to the original map, and divided this result by two to obtain the final probabilistic map. **B** The group-level functional reference map. We set the threshold of probabilistic map to 0.2, and labeled the corresponding large clusters as specific brain areas, including the aIPS, pIPS, insula, IFG, ITG, PM, MOG, and ACC

To generate the group-level functional reference map, we first averaged the binary activation map (threshold: z > 1.65) of all participants to obtain the averaged probabilistic map. To avoid any asymmetry in brain areas between hemispheres, we flipped the averaged probabilistic map, added it to the original map, and divided this result by two to obtain the final probabilistic map (Fig. 3A). Then, we applied a threshold of 0.2 to the probabilistic map and used the watershed algorithm to segment the activation areas, through which 23 clusters were obtained. Finally, four small clusters (with volume size smaller than 100 voxels) were discarded, and the remaining 19 large clusters were labeled as ROIs to acquire the group-level functional reference map (Fig. 3B). These clusters were then labeled according to the Automated Anatomical labeling (AAL3) template (Rolls et al. 2020; Wang et al. 2015) and probabilistic maps of visual topography (Rolls et al. 2020; Wang et al. 2015). Moreover, for analysis convenience, we collapsed the ACCs in both hemispheres into one cluster, labeled as bilateral ACC. The FreeROI tool was used to delineate ROIs in this procedure (Zhen et al. 2015).

### Functional probabilistic atlas

To quantify individual variability in the numerosity-related brain regions, a functional probabilistic atlas was constructed based on subject-specific ROIs. To do this, subject-specific ROIs were binarized and then averaged across participants to obtain the probability of activation in each region. This probability index reflected the likelihood of activation of a particular ROI at a given voxel across participants, which allowed us to obtain a voxel-based description of inter-individual variability in neural activation during a numerosity task. Finally, to construct the functional probabilistic atlas, we compared the activation probability of each ROI in each voxel. A voxel was assigned to the ROI with the maximum activation probability at that location. Any voxel with a maximum probability of less than 20% was set to 0%, as it may not belong to any ROI. By doing so, the functional probabilistic atlas for numerosity processing was created, providing non-overlapping brain map that characterized activation probability across participants. This functional probabilistic atlas will be a reliable tool for investigating inter-individual differences in neural activities.

### Individual, hemispheric, and sex differences

Using these subject-specific ROIs, we can extract information about brain activation such as activation volume, magnitude, and peak value coordinates. Activation volume and peak value coordinates could be calculated directly from the ROIs in the MNI reference system. For activation magnitude, in line with previous studies (Lipkin et al. 2022; Fedorenko et al. 2010), we defined it as the percentage of signal change (PSC) of the contrast between numerosity comparison and luminance comparison conditions. In the GLM model, we obtained the estimated parameter β of the contrast of each voxel. We then divided β by the GLM intercept to obtain the PSC. The average PSC of activated voxels in a brain area was considered as the activation magnitude of that area. To assess inter-individual differences in brain activation, we calculated the mean and standard deviation of the activation volume, magnitude and peak coordinates across participants.

Next, we examined hemispheric differences and sex differences in these subject-specific ROIs. For activation volume and activation magnitude, we conducted paired two-sample t-tests to test the differences of the corresponding brain areas in different hemispheres. For peak value coordinates, we employed one-sample t-tests on the difference values of right and left areas. Since the x-axis is symmetric in right and left, the difference values were calculated by absolute values of the right areas minus absolute values of the corresponding left areas. For the y-axis and z-axis, the difference values were calculated directly from the coordinates of the right minus the coordinates of the left. Higher difference values correspond to more lateral, anterior and superior locations of the activation peak. All t-tests were corrected with FDR correction (adjusted *p* < 0.05).

Similarly, to explore potential sex differences in all ROIs, we measured activation volume and magnitude in male and female groups. Independent two-sample t-tests was used to examine the significance of sex differences, and multiple comparisons were corrected with FDR correction (adjusted *p* < 0.05).

### Correlations between brain activations and behavioral performances

To prove the usability of the numerosity probabilistic atlas, we calculated the correlations between brain activation in each ROI and the behavioral performance. Behavior performance was calculated by computing the residual of accuracy and RT in a regression model where number comparison condition was the dependent variable and luminance comparison condition was the independent variable, which has been demonstrated to be effective in removing variance associated with the control condition (DeGutis et al. 2013). Activation magnitude was measured by the averaged z-score for the contrast of number comparison versus luminance comparison in each region, which can be drawn from each subject’s activation image and ROI. Among the 456 participants, 25 of them were further excluded from the correlation analysis because of their behavioral accuracies or RTs being more than three interquartile or standard deviations away from the group mean. To ensure the reliability of our results, we randomly assigned the remaining 431 subjects into two groups, one with 216 subjects and the other with 215 subjects. We only considered a brain regions’ correlation coefficient to be significant if it was found to be significant in both groups.

### Evaluation of functional probabilistic atlas

Finally, we evaluated the reliability of our functional probabilistic atlas by examining four features of the atlas: anatomical correspondence (Figure S2), inter-rater reliability (Figure S3), sample size effect (Figure S4), similarity between meta-analytic maps (Figure S5) (see Supplementary Materials for details).

## Results

### Behavioral results

The accuracy rates were higher than 90% in both tasks, indicating that the subjects were attentive and performed the tasks accurately. Table 1 presents the behavioral results of two tasks. There was a significant difference in the accuracies between the numerosity and luminance tasks (t(430) = − 24.524, *p* < 0.001) (see Supplementary materials for more details).

**Table 1:** Behavioral results of numerosity and luminance comparison tasks

Task condition	Mean (SD)
Numerosity comparison	**0.900 (0.059)**
Luminance comparison	**0.967 (0.040)**

### Creating functional probabilistic atlas

**Fig. 4:**
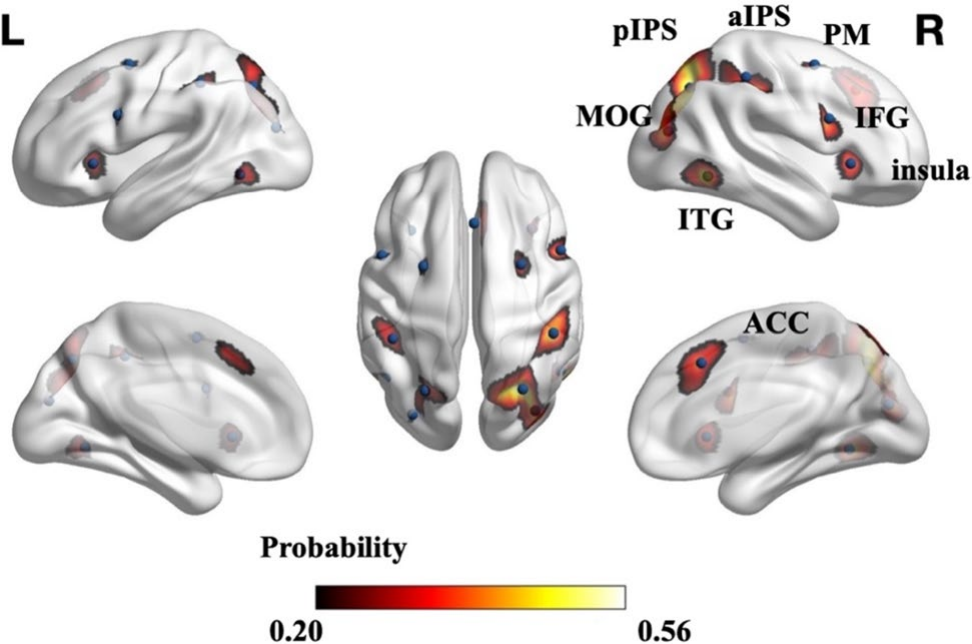
Functional probabilistic atlas of numerosity processing (*p* > 0.2). The Value in each voxel represents the activation probability, which measures the likelihood that a given ROI is activated at a given voxel across participants. Any value below 0.2 was assigned a value of zero. The blue points on the figure represent the averaged peak coordinates of each numerosity-related ROI

During the numerosity localizer task, eight functional regions were consistently activated across individuals in each hemisphere, including the ITG, aIPS, pIPS, PM, insula, IFG, MOG, and ACC. As shown in Fig. 4, a functional probabilistic atlas of numerosity was created. The average peak coordinate of every functional region was also displayed in the atlas. Notably, the peak coordinates tended to locate in regions with higher probability.

Table 2 summarizes various measures that were used to characterize the functional probabilistic atlas, including the maximum probability and the percentage of subjects for a given ROI. The percent of subjects in each ROI ranged from 54.82% (L PM) to 95.18% (R pIPS), indicating that not all brain regions were equally involved in the numerosity comparison condition. The bilateral ITG, aIPS, pIPS, ACC, and right MOG were more consistently activated during numerosity comparison, ranging from 85.09% to 95.18%, while the bilateral PM, insula, IFG, and left MOG were less consistently activated, ranging from 54.82% to 80.48%. Another measure was the maximal activation probability of voxels within each functional region, which was found to be around 35%. This observation suggested significant individual differences in neural activation during numerosity processing. The regions with the highest probability of activation were the right pIPS (56.80%) and right ITG (52.63%), while the left PM (23.90%) and left IFG (25.88%) showed the lowest probability. These findings demonstrated the marked inter-individual variability in neural activation across various brain regions during numerosity processing.

**Table 2:** Characterizations of numerosity-related functional regions: percent of subjects, maximum probability, activation volume in cm^3^, PSC, and MNI peak coordinates

ROI	Percent subjects	Maximum probability	Volume (cm^3^) Mean (SD)	PSC Mean (SD)	MNI coordinates Mean (SD)		
					X	Y	Z
R ITG	90.35	52.63	3.96 (3.79)	0.33 (0.09)	50 (5)	− 57 (6)	− 7 (4)
L ITG	81.36	32.68	2.54 (2.61)	0.28 (0.07)	− 47 (6)	− 61 (8)	− 6 (5)
R MOG	85.53	42.54	3.04 (2.94)	0.27 (0.06)	36 (5)	− 79 (7)	18 (6)
L MOG	68.86	29.17	1.88 (2.23)	0.26 (0.06)	− 32 (6)	− 81 (8)	20 (5)
R aIPS	90.13	47.59	6.64 (5.99)	0.40 (0.13)	46 (8)	− 36 (8)	48 (8)
L aIPS	85.96	36.62	3.99 (4.35)	0.30 (0.10)	− 43 (7)	− 39 (7)	45 (8)
R pIPS	95.18	56.80	9.90 (8.18)	0.40 (0.12)	29 (6)	− 67 (8)	42 (11)
L pIPS	86.18	38.16	5.48 (5.66)	0.36 (0.12)	− 25 (7)	− 68 (10)	43 (12)
R PM	64.47	31.14	2.98 (3.04)	0.29 (0.09)	28 (6)	2 (7)	55 (6)
L PM	54.82	23.90	2.49 (2.69)	0.27 (0.08)	− 26 (5)	1 (7)	55 (6)
R insula	71.49	39.04	2.50 (2.79)	0.25 (0.07)	34 (4)	22 (5)	0 (7)
L insula	69.30	32.68	2.25 (2.52)	0.27 (0.07)	− 33 (5)	21 (8)	0 (8)
R IFG	80.48	43.64	3.31 (3.28)	0.32 (0.08)	50 (9)	10 (4)	25 (8)
L IFG	61.18	25.88	2.59 (2.75)	0.32 (0.09)	− 49 (8)	7 (6)	27 (8)
BI ACC	85.09	43.20	5.87 (5.92)	0.31 (0.07)	2 (6)	25 (8)	41 (9)

*ITG*, inferior temporal gyrus, *MOG* middle occipital gyrus, *aIPS* anterior interparietal sulcus, *pIPS* posterior interparietal sulcus, *PM* premotor area, insula, *IFG* inferior frontal gyrus, *ACC*, anterior cingulate cortex, *L* left hemisphere, *R* right hemisphere *BI* bilateral

### Inter-individual differences

**Fig. 5:**
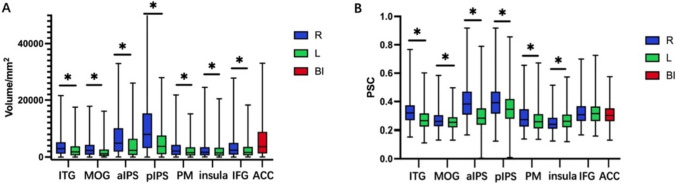
Distributions of activation volume and magnitude in the numerosity-related regions. **A** Box-plot of activation volume across individuals. **B** Box-plot of PSC across individuals. L, left hemisphere; R, right hemisphere; BI, bilateral. The upper and lower limits of the box represent the maximum and minimum values, respectively, and the three black dash lines within the box from top to down indicate the first quartile (25%), median (50%), and third quartile (75%). The asterisk above the short-dash line represents the significance of difference between the two hemispheres. *, *p* < 0.05. Multiple comparison was corrected by FDR correction (Adjusted *p* < 0.05)

The mean and standard deviation (SD) of activation volume and magnitude were shown in Table 2, while the box-plots in Fig. 5 provided more detailed information. Among these ROIs, the right pIPS exhibited the largest volume (average: 9.90 cm^3^), while the left MOG had the smallest volume (average: 1.88 cm^3^). For the activation magnitude, the right aIPS and the right pIPS showed the highest PSC (average: 0.40 for both clusters), while the right insula exhibited the lowest PSC (average: 0.25). These results demonstrated significant inter-individual differences in both activation volume and magnitude across the numerosity-related brain regions, as illustrated in Fig. 5A, B.

**Fig. 6:**
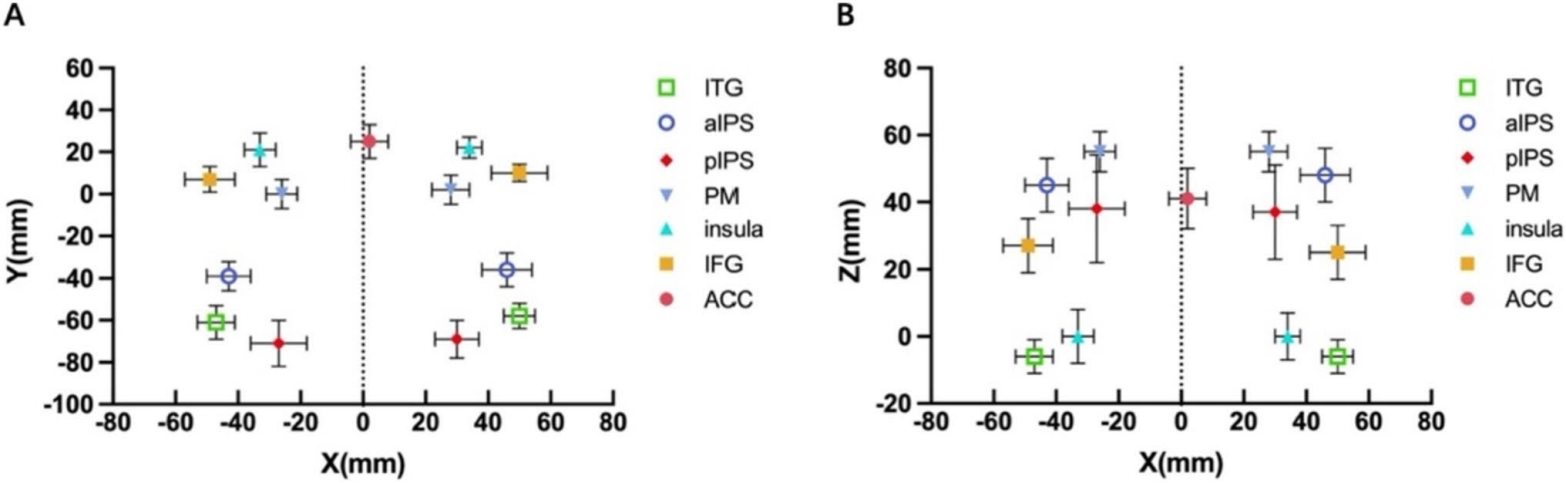
Distributions and variability of spatial location in numerosity-related regions**.** (A, B) Mean coordinates and standard deviations (SD) of peak activations across individuals in the left and right hemispheres. Left–right, X axis; Anterior–posterior, Y axis; Superior-inferior, Z axis. For regions in the left hemisphere, Left–right coordinates were less than 0, and for regions in the right hemisphere, left–right coordinates were greater than 0

Then, to investigate inter-individual differences in the spatial location of these brain activations, we calculated the mean and SD of the peak activation coordinates in each region along the x, y, and z axes (Table 2). The SDs in most regions were around 8 mm across participants, indicating that inter-individual differences in spatial location commonly existed in numerosity-related regions. Individual variabilities in the Y and Z axes were generally higher in the bilateral pIPS and aIPS. Figures 6A, B displayed the individual variability in peak activation coordinates, accompanied by their respective SDs.

### Hemispheric differences

Three measures were used to assess hemispheric differences in numerosity-related activation: activation volume, magnitude, and peak value coordinates.

First, several regions showed significant differences in activation volume and magnitude between the right and left hemispheres. As shown in Fig. 5A, all regions in the right hemisphere had significantly larger volumes than their counterparts in the left hemisphere (IFG: t(256) = 7.028, *p* < 0.001; insula: t(286) = 2.728, *p* = 0.007; pIPS: t(389) = 17.966, *p* < 0.001; ITG: t(353) = 10.350, *p* < 0.001; PM: t(220) = 4.807, *p* < 0.001; aIPS: t(372) = 13.048, *p* < 0.001; MOG: t(291) = 9.058, *p* < 0.001; after FDR correction). Similarly, most regions also showed significant hemispheric asymmetries in activation magnitudes, except for IFG (Fig. 5B). Specifically, the pIPS, ITG, PM, and aIPS exhibited stronger activation in the right hemisphere (pIPS: t(388) = 9.761, *p* < 0.001; ITG: t(352) = 13.914, *p* < 0.001; PM: t(219) = 4.639, *p* < 0.001; aIPS: t(370) = 17.064, *p* < 0.001; MOG: t(288) = 4.465, *p* < 0.001; after FDR correction), while the insula showed leftward asymmetry (insula: t(284) = − 4.103, *p* < 0.001; after FDR correction).

**Fig. 7:**
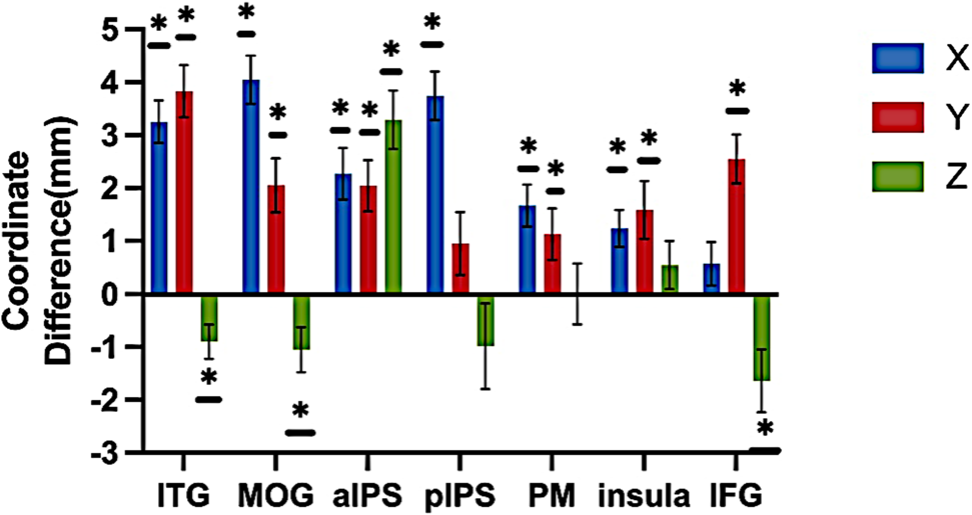
The asymmetry of coordinates in each region. Positive coordinate difference in x, y, or z axes indicated the ROI in right hemisphere located in more lateral, anterior, or dorsal position than its counterpart in the left hemisphere. Multiple comparisons were corrected with FDR correction (adjusted *p* < 0.05). Error bars indicate standard error of mean. ∗ , *p* < 0.05

Furthermore, to investigate hemispheric differences in spatial locations, we calculated the coordinate differences between the right and left hemispheres and conducted one-sample t-tests on the difference of x, y, and z axes in each region. Our results were shown in Fig. 7. The majority of asymmetries were associated with the x and y axes compared to the z-axis. Specifically, for the x-axis, ITG, MOG, aIPS, pIPS, PM, and insula exhibited significant positive differences (ITG: t(353) = 8.112, *p* < 0.001; MOG: t(291) = 8.857, *p* < 0.001; aIPS: t(372) = 4.683, *p* < 0.001; pIPS: t(389) = 8.132, *p* < 0.001; PM: t(220) = 4.217, *p* < 0.001; insula: t(286) = 3.554, *p* < 0.001; after FDR correction), indicating that these regions in the right hemisphere located more laterally than their counterparts in the left hemisphere. For the y-axis, ITG, MOG, aIPS, PM, insula, and IFG showed significant positive differences (ITG: t(353) = 7.749, *p* < 0.001; MOG: t(291) = 4.026, *p* < 0.001; aIPS: t(372) = 4.240, *p* < 0.001; PM: t(220) = 2.334, *p* = 0.025; insula: t(286) = 2.893, *p* = 0.006; IFG: t(256) = 5.476, *p* < 0.001; after FDR correction), indicating that these regions were more anteriorly activated in the right than in the left hemisphere. For the z-axis, only four regions showed hemispheric differences (ITG: t(353) = − 2.767, *p* = 0.016; MOG: t(291) = − 2.445, p = 0.026; aIPS: t(372) = 5.978, *p* < 0.001; IFG: t(256) = − 2.743, *p* = 0.016; after FDR correction). Specifically, the right aIPS was located in a more dorsal area compared to the left, while the right ITG, MOG, and IFG were positioned more ventrally. Taken together, our results showed variability in spatial location between the two hemispheres in all regions, but this varied among the axes.

### Sex differences

**Fig. 8:**
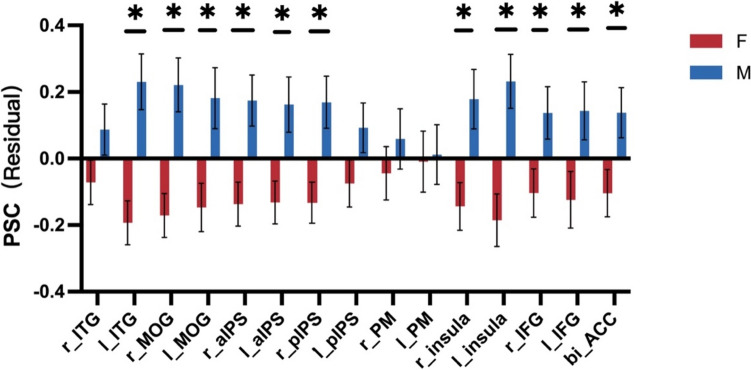
Differences in activation magnitude between males and females in numerosity-related regions. An independent two-sample t-test was used to assess the difference in the regression residual of activation magnitude between males and females. Multiple comparisons were corrected using the FDR correction (adjusted *p *< 0.05). Error bars indicate standard error of mean. *, *p* < 0.05

There was no significant difference between males and females in the behavioral performance of numerosity task (t(429) = 0.040, *p* = 0.968). We next investigated potential sex differences in activation volume, magnitude, and peak value coordinates during numerosity processing, using an independent two-sample t-test. After correcting for multiple comparisons using the FDR correction, our results indicated no significant sex differences in peak value coordinates and activation volume. However, we found significant sex differences in activation magnitude. Specifically, males demonstrated stronger activation than females in multiple brain regions, such as left ITG, bilateral MOG, bilateral aIPS, right pIPS, bilateral insula, bilateral IFG, and the ACC, even after controlling for gray matter volume (GMV) differences between the sexes in each respective region (L ITG, t(347) = − 4.031, *p* = 0.001; R MOG, t(365) = − 3.799, *p* = 0.001; L MOG, t(293) = − 2.843, *p* = 0.009; R aIPS, t(389) = − 3.092, *p* = 0.007; L aIPS, t(368) = − 2.845, *p* = 0.009; R pIPS, t(410) = − 3.077, p = 0.007; R insula, t(307) = − 2.852, *p* = 0.009; L insula, t(295) = − 3.652, *p* = 0.002; R IFG, t(344) = − 2.235, *p* = 0.04; L IFG, t(263) = − 2.191, *p* = 0.04; BI ACC, t(365) = − 2.312, *p* = 0.04; after FDR correction) (Fig. 8) (Supplementary materials). In sum, our findings suggested that males and females exhibited similar brain activation patterns in terms of activation volume and peak value coordinates during numerosity processing, but males tended to exhibit stronger activation in certain brain regions than females.

### Correlations between brain activations and behavioral performances

**Fig. 9:**
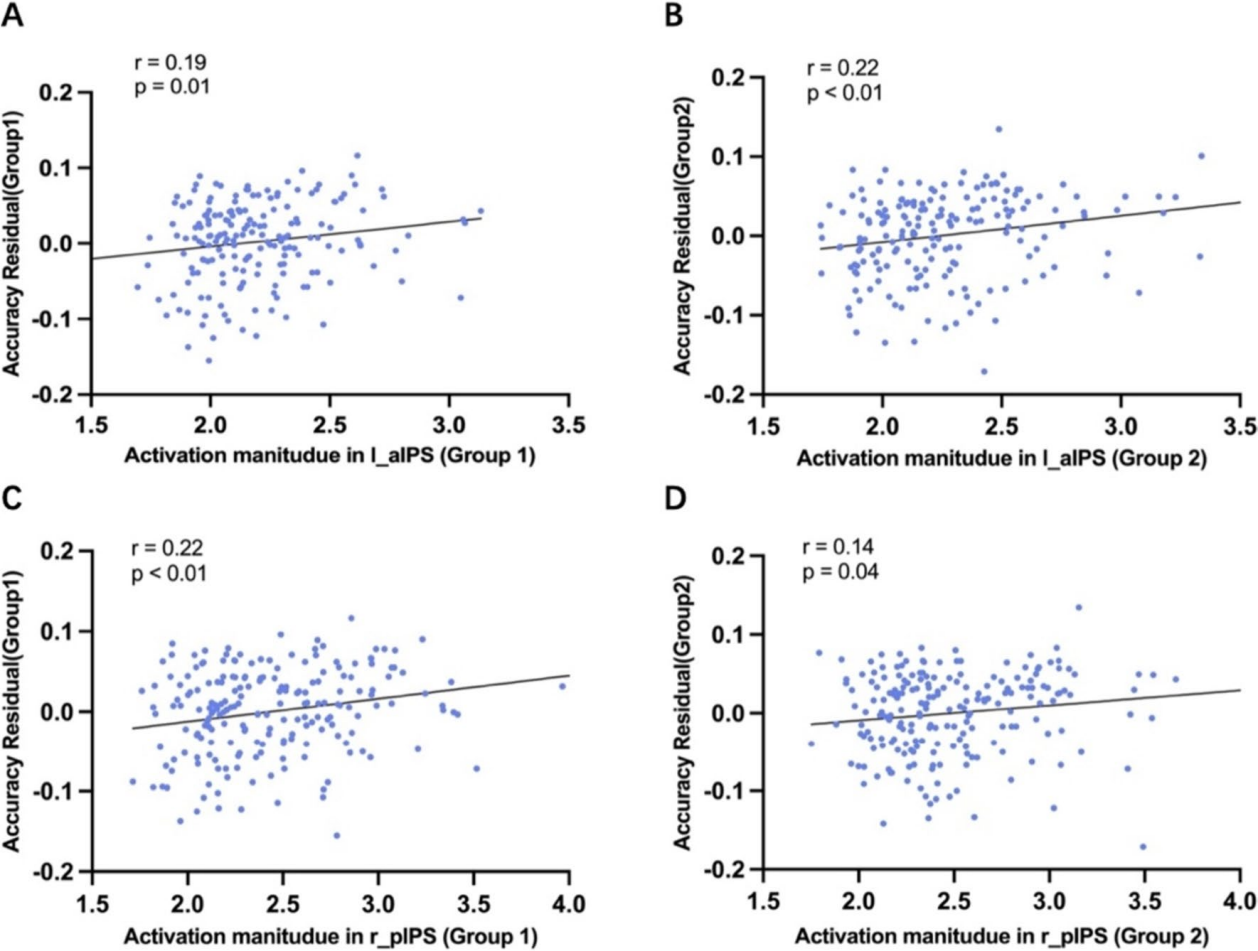
Correlations between brain activations and behavioral performance in left aIPS and right pIPS. The activations were calculated as the averaged z-score with the contrast of numerosity comparison versus luminance comparison. Behavioral performance of the Numerosity comparison condition was calculated as the residual of accuracy, which was obtained by regressing accuracy in the luminance comparison task from that in the numerosity comparison task. Participants were randomly divided into two groups (Group 1: *N* = 216; Group 2: *N* = 215)

To explore the link between brain activity and behavior, we randomly divided the participants into two groups and examined the correlation between activation magnitudes in specific brain regions and the residual accuracy of the numerosity comparison task for both groups. The results showed that only the activation magnitudes in two regions, i.e., left aIPS and right pIPS, were positively correlated with the residuals of behavioral accuracy in both groups (Group 1: left aIPS: *r* = 0.19, *p* = 0.01; right pIPS: *r* = 0.22, *p* < 0.01; Group 2: left aIPS: *r* = 0.22, *p* < 0.01; right pIPS: r = 0.14, p = 0.04; Fig. 9A–D). There were also some regions showed significant correlation in only one group (Group 1: left ITG: *r* = 0.17, *p* = 0.03; right PM: *r* = 0.19, *p* = 0.03; ACC: *r* = 0.16, *p* = 0.03; Group 2: right ITG: *r* = 0.15, *p* = 0.04; left pIPS: *r* = 0.25, *p* < 0.01; left PM: r = 0.19, *p* = 0.04; left IFG: r = 0.18, *p* = 0.04). In the remaining regions, correlations were not significant in either group (all rs < 0.15, all ps > 0.05). These findings provided strong evidence for a strong association between neural activation and behavioral performance, supporting the utility of our functional probabilistic atlas. Moreover, it also suggested that our atlas could serve as a reliable and robust functional and spatial reference for standardization of functional localization in future fMRI investigations.

## Discussion

The goal of the present study is to quantify the inter-individual variability of neural activations associated with the representation and processing of numerosity information and establish a functional probabilistic atlas for numerosity-related neural processing. Using a large-sample fMRI dataset, we identified several subject-specific ROIs and these ROIs showed considerable individual, hemispheric, and sex differences in the location of peak activation, activation volume, and activation magnitude. Our functional probabilistic atlas aligned well with the meta-analytical map of numerosity created from Neurosynth (Yarkoni et al. 2011) (Supplementary materials; Fig. S5), and the correlation between activation magnitude and behavioral performance in the left aIPS and right pIPS demonstrated the practicality and feasibility of our atlas. Thus, the functional probabilistic atlas offers a robust spatial reference for the functional localization of numerosity-related neural network.

Our study diverged from earlier studies in two significant aspects. First, while most existing neuroimaging studies relied on group-averaged or anatomically defined ROIs and thus neglected individual variability, our study identified subject-specific activation areas, which allow us to systematically quantify individual differences in numerosity-related brain activities. Second, we advocated for larger sample sizes for more accurate ROI definition, addressing a frequent limitation of previous studies, as evidenced in Figure S5. Although meta-analyses have been used to mitigate the limitations imposed by small sample sizes (Cohenkadosh et al. 2008; Sokolowski et al. 2017), these approaches focused mainly on the spatial and functional consistency of neural activations across individuals, still neglecting individual variations. Therefore, our study enriched existing research by characterizing the inter-individual variability in brain activations within well-defined numerosity-related regions. These regions, including the bilateral ITG, MOG, aIPS, pIPS, PM, insula, IFG, and ACC, aligned with previously identified brain areas in the literature (Piazza et al. 2007; Piazza and Izard 2009; Holloway et al. 2010; Vogel et al. 2013; Hayashi et al. 2013; Leibovich et al. 2016; Li et al. 2019; Pinheiro-Chagas et al. 2018; Ustun et al. 2021). Such interindividual variability likely provided a neural basis for interindividual differences in numerosity perception. For instance, individual differences in functional connectivity within a distributed numerosity-related brain network could predict individual differences in the acuity of Approximate Number System (ANS) in behavior (Zhang et al. 2023).

First, in our study, we revealed substantial differences among individuals in both the functional and spatial characteristics of these numerosity-related ROIs. In general, the interindividual variability in functionally specialized cortical regions can be traced back to a combination of genetic, structural, and environmental influences during development, including variations in cytoarchitecture (e.g., Amunts et al. 1999; Caspers et al. 2013; Reardon et al. 2018), neural connectivity patterns (e.g., Passingham et al. 2002; Mueller et al. 2013; Smith et al. 2015), cortical competition (e.g., Dehaene 2013; Golarai et al. 2007; Sporns 2011; Zhao et al. 2008), and genetic factors (e.g., Hawrylycz et al. 2015). Collectively, these factors may contribute to the observed interindividual variability in brain activations related to numerosity perception. Importantly for our study, existing evidence indicated that the adolescents and adults engaged different brain regions when processing non-symbolic numerical information, and these regions undergo substantial age-related modifications (e.g., Ansari and Dhital 2006; Cantlon et al. 2006; Kaufmann et al. 2011). These findings highlighted the influence of developmental factors in shaping the neural substrates underlying non-symbolic numerosity processing, and suggested that these developmental changes could contribute to the interindividual variability we observed in numerosity-related ROIs.

Moreover, cognitive strategies employed during non-symbolic number processing might also account for the variability. Previous research has demonstrated that individuals may adopt different cognitive strategies when performing dot estimation tasks, and different cognitive strategies activated diverse brain areas. For example, Gandini et al. (2008) found that different strategies for approximate quantification during a numerosity estimation task led to the activation of distinct neural networks in young adults. Likewise, Moscoso et al. (2022) reported that numerosity estimation strategies for grouped and ungrouped stimuli engaged both common and distinct areas within the frontoparietal network. These findings, along with the sex differences elaborated upon later, may not only help reconcile disparate findings mentioned earlier in the introduction—specifically, the various brain regions previously implicated in numerosity perception—but also contribute to the observed variability in numerosity-related brain regions. Therefore, both developmental changes and strategy-based differences could be factors contributing to the interindividual variability in numerosity-related ROIs.

Interestingly, the degree of interindividual variability in brain activations differed across regions. As shown in Table 1, the bilateral IPS, particularly the right IPS, was consistently activated in more than 85% of participants and exhibited more substantial variations in activation magnitude and peak activation coordinates, in contrast to other regions which showed less consistency and smaller variations. This finding aligned with the work of Haist et al. (2015), which reported a significant developmental trajectory for improved numerosity precision across a range of brain regions, including the bilateral IPS, AG, SMG, SPL, and precuneus, as well as the left IFG and right MFG. Similarly, the right parietal cortex, especially the IPS, exhibited the most pronounced age-related effects, which may potentially account for its higher variability in activation patterns. Building on these findings, we hypothesized that the consistent activation and high variability within the IPS within the IPS may underlie its crucial role in supporting a numerical representation that is explicitly read out for numerical decisions and behavior (Eger et al. 2009; Harvey et al. 2013; Lasne et al. 2019; Piazza et al. 2004; Dormal and Pesenti 2009; Dormal et al. 2012; Lecce et al. 2015; Kersey and Cantlon 2017). Corroborating this hypothesis, we found that although all ROIs demonstrated some degree of interindividual variability, it was the variability in the activation magnitude of the right pIPS that significantly correlated with numerosity discrimination performance.

Second, we found significant hemisphere difference in these numerosity-related ROIs. For the location of peak activation and activation volume measures, regions in the right hemisphere were generally larger and located more lateral and anterior compared to their counterparts in the left hemisphere. The reason of the right asymmetry in numerosity-related regions remains largely unknown. One possibility is that, the right hemisphere mainly processes spatially non-symbolic information whereas the left hemisphere dominantly processes symbolic information (Hill et al. 2014; Braver et al. 2001). In our study, the test stimuli contained randomly distributed dots that provides non-symbolic number information as well as spatial information such as shape and location. Consistent with previous studies using similar non-symbolic stimuli (Piazza et al. 2006; Cohenkadosh et al. 2008; Dormal and Pesenti 2009; Kadosh et al. 2007), the brain activations in most regions showed right lateralization. In contrast, previous studies on symbolic numerosity, such as Arabic numbers, have shown stronger activation in the left hemisphere (Kadosh et al. 2007; Ansari 2007; Notebaert et al. 2011). For example, Notebaert et al. (2011) found that habituation and de-habituation of Arabic numbers only occurred in the left parietal cortex, indicating the left brain plays a more significant role in symbolic numerosity processing. Therefore, our findings provided new evidence, from the individual difference perspective, supporting the right-biased hemispheric asymmetry in non-symbolic numerosity processing. However, for the activation magnitude, although aIPS, pIPS, PM, MOG, and ITG showed a similar right-biased asymmetry, the insula showed a left-biased asymmetry and IFG showed no obvious asymmetry. Previous research has suggested that the accurate estimation of the numerosity in dot displays depended not only on the processing of numerosity information, but also on the ability to ignore irrelevant features interfering with the numerosity information (e.g., non-numerical magnitude properties), which may rely on inhibitory functions in the brain (Szucs et al. 2013). Thus, we speculated that the IFG and insula may be responsible for the function of execution control in numerosity processing (i.e., inhibiting the irrelevant non-numerical magnitude information), which is known to be lateralized to the left (Silva et al. 2014; John 1973).

Third, it is noteworthy that we observed significant sex differences in brain activations within these numerosity-related ROIs, even when no behavioral differences were evident between males and females. However, unlike the hemispheric differences mentioned above, sex differences were only found in the activation magnitude between males and females. Males generally had stronger activation than females in most ROIs, except for the right ITG, left pIPS, and bilateral PM. We proposed several tentative explanations for these findings. First, the behavioral tasks may not be sensitive enough to capture nuanced sex differences, whereas neuroimaging techniques like fMRI provided a more granular perspective. Second, anatomical differences in brain structure could contribute to these findings (e.g., Liu et al. 2020; Ritchie et al 2018). For instance, Keller and Menon (2009) observed sex differences in brain activity patterns during mathematical cognition tasks. They argued that these differences may be attributed to underlying anatomical differences between males and females. Females had greater regional density and volume in several posterior brain areas where functional activation differences were detected between males and females. Specifically, females showed greater density and volume in the intraparietal sulcus, parahippocampal gyrus, extrastriate cortex, supramarginal gyrus, angular gyrus, superior parietal lobule, and inferior temporal cortex. On the other hand, males did not show greater density or volume in any brain region. They concluded that the structural differences in these brain regions may contribute to the observed sex differences in brain activity patterns during mathematical tasks. However, this factor alone cannot account for our findings as we found that, after controlling for GMV variations between males and females, significant sex differences still persisted in most numerosity-related brain areas. Third, hormonal differences between males and females may also play a role in modulating cognitive function and the development of brain structure (e.g., Wallen 2009; Ritchie et al 2018). These hormones interact with neural circuits, potentially leading to sex-specific variations in brain activation patterns. For instance, Pletzer et al. (2011) revealed that sex differences in brain activations during a number bisection task were influenced by the menstrual cycle phase. Specifically, differences between males and females were more pronounced during the follicular phase and diminished during the luteal phase, particularly in regions like the medial prefrontal cortex and inferior parietal lobules. Although their findings focused on the symbolic number processing, it is reasonable to speculate that the hormonal differences might lead to the sex differences observed in the current study if considering the close relationship between symbolic and non-symbolic number representations (Carey 2011; Lipton and Spelke 2005; Dehaene 2011). Fourth, Cognitive strategies might differ between males and females, activating distinct neural circuits. These neural differences may not manifest as observable behavioral differences but could be detectable at the neural level. Although previous studies have seldom addressed the sex differences in the neural circuits underlying non-symbolic number perception, there was evidence showing that males and females recruited differential neural networks during a multi-digit number comparison task involving the symbolic numbers, and these differences were attributable to the use of different cognitive strategies by males and females (Pletzer et al. 2013). Pletzer (2016) further explored sex differences in arithmetic operations and found that males exhibited distinct neural systems for subtraction and multiplication, while these systems were largely overlapped in females, suggesting significant sex differences in brain activations associated with arithmetic operations. They argued that females may employ different neural substrates and prefer verbal and memory strategies over spatial ones compared to men to achieve similar behavioral performance, or that females might use a broader range of strategies, leading to more varied brain activation patterns. In contrast, Chen and Chang (2023) also observed sex-related patterns in the brain regions associated with arithmetic, including the left middle frontal gyrus (MFG), left intraparietal sulcus (IPS), and insula. These regions showed substantial brain responses to problem size effects in females, while males showed marginal effects. They speculated that females may rely more on algorithmic calculations and procedural strategies, while males may use faster rote-fact retrieval, estimation, and insight strategies during mathematical problem-solving. Despite of these inconsistent findings in brain activity patterns between males and females, the prevailing explanation for the sex differences has been attributed to the use of different cognitive strategies by men and women, thereby activating different brain regions (Chang et al. 2022). Given the close relationship between non-symbolic and symbolic number perception and mathematic performance (Mazzocco et al. 2011; Libertus et al. 2011; Starr et al. 2013; Tibber et al. 2012; Anobile et al. 2016, 2018), it is plausible that the observed sex differences in non-symbolic number perception could contribute to sex differences in certain high-level mathematical abilities or, at the least, these abilities may covary. Further research is needed to explore all these possibilities more thoroughly.

Finally, our study makes another significant contribution by introducing a novel functional probabilistic atlas for exploring the neural mechanisms underlying numerosity processing, which can offer several advantages. First, it may act as a quantitative spatial reference system for integrating information from multiple imaging modalities, e.g., structural, connectional, and even molecular variations in functional regions, to provide a comprehensive understanding of the neurobiological basis of numerosity processing. Second, the probabilistic atlas could facilitate fMRI research by providing subject-specific functional ROIs for group studies or as prior constraints in individual studies. At last, the probabilistic atlas can offer a more accurate reference for clinical research, allowing for the detection of any deficits in patients and quantifying deviations from healthy controls, compared to a deterministic atlas.

It should be noted that there are several potential limitations of our study. First, our participants were only college students. Thus, the validity of our findings should be confirmed in other adult populations and our findings may not be generalized to adolescents and the elderly. Future studies could include participants of different ages to examine the development of brain activity related to numerosity. Second, given that the dots in the stimuli were of uniform size, we cannot completely rule out the possibility that total surface area of the dot array may also contribute the observed brain activations. Future investigations with more refined design should be employed to eliminate this potential confound. Third, our study adopted only visual dot arrays as stimuli. To achieve a comprehensive understanding of the individual variability in supramodal numerosity processing, it's essential to include numerosity information from haptic and auditory stimuli and examine the similarities and differences between different modalities.

## Conclusion

Our study thoroughly characterized the individual, hemispheric, and sex differences in brain activations during numerosity processing, offering a new perspective on inter-individual variability in the neural substrates underlying the representation and processing of numerosity information. Additionally, we developed a functional probabilistic atlas based on a large sample dataset, which can serve as a reliable functional and spatial reference for standardizing functional localization and facilitate future neuroimaging investigations.

### Supplementary Information

Below is the link to the electronic supplementary material.

Supplementary file1 (DOCX 1544 KB)

## Data Availability

The probabilistic atlas that supports the findings of this study is openly available at http://www.brainactivityatlas.org. Custom code and imaging data can be accessed on GitHub at https://github.com/zangzhongyao/Data-and-code-.git. Raw data is available via a request to the corresponding author (husiyuan@bnu.edu.cn) with the need for a formal data-sharing agreement.
