# Supplementary material for: Inter-individual, hemispheric and sex variability of brain activations during numerosity processing
Source: Brain Struct Funct. 2024 Jan 10;229(2):459–75. doi: 10.1007/s00429-023-02747-3 (PMC10917853; doi:10.1007/s00429-023-02747-3)
Supplement: Supplementary file 1 — Supplementary file1 (DOCX 1544 KB) [file 429_2023_2747_MOESM1_ESM.docx]

**Supplementary materials**

**Numerosity localizer task**

A numerosity localizer task was used to identify brain areas associated with numerosity processing. The stimuli for this task consisted of images containing cyan dots. The dot arrays had six different numerosity levels (6, 7, 8, 9, 10, and 12), and the dots' lightness, represented in RGB values, had five levels (level 1: [67, 151, 152]; level 2: [80, 151, 152]; level 3: [92, 201, 202]; level 4: [105, 227, 229]; level 5: [117, 251, 253]). Taking into account both number and lightness, we generated 30 variations of dot images with controlled convex hull and density. Each variation comprised 40 images, which were presented randomly during the task.

For each numerosity comparison trial, five scales were used to represent the number ratio between the two dot images (6:12, 9:12, 10:12, 7:8, and 9:10). To verify that the convex hull and density were controlled as intended, we extracted relevant data from all numerosity comparison trials across all subjects. We then grouped the data into five categories based on the scales, removed duplicate rows, and conducted paired sample t-tests between the small and large numbers (e.g., in the 6:12 scale, 6 is the small number and 12 is the large number). Convex hull areas were determined using the MATLAB function of convhull, and density was calculated by dividing the number by the corresponding convex hull areas. The results are displayed in Figure S1. Most of the convex hull areas and densities did not show significant differences in the tests (convex hull: all ps > 0.2; density: all ps > 0.4), with the exception of the convex hull areas in the 6:12 scale (t(329) = -2.313, p = 0.021). This suggests that, in the majority of trials, the convex hull and density were effectively controlled.

*
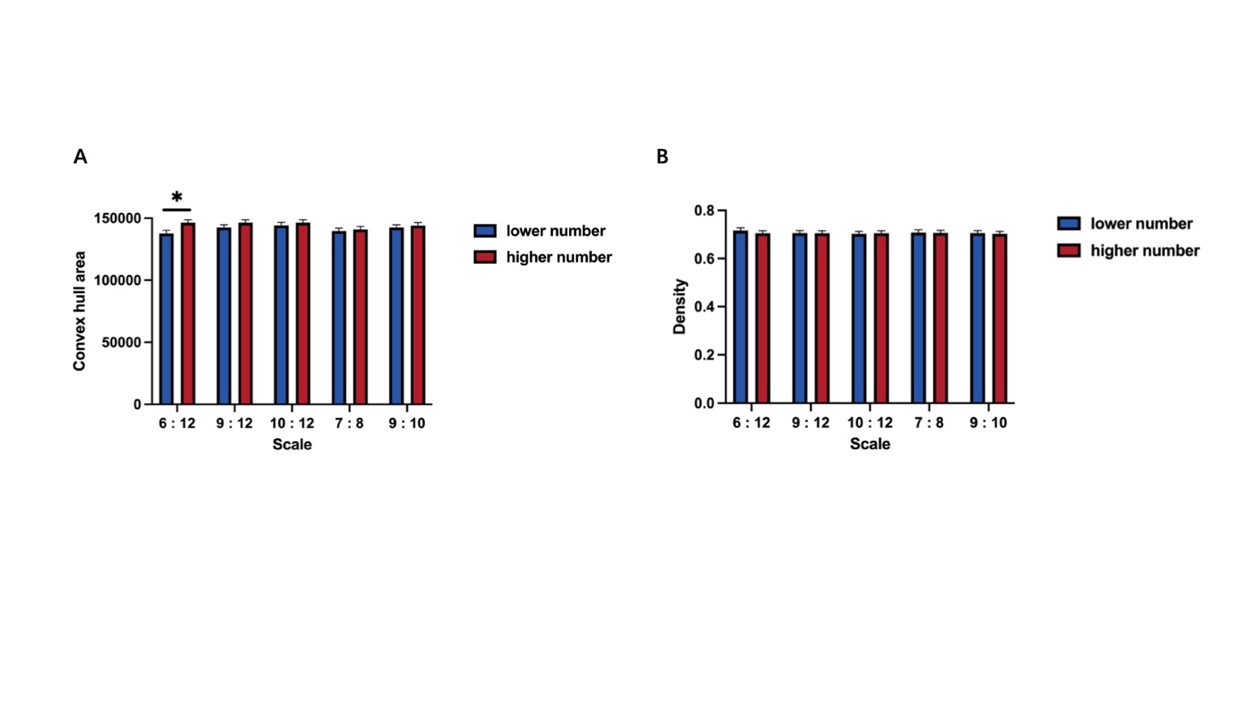
*

**Figure S1. Convex hull areas and densities in different scales.** Error bars indicate standard error of the mean.

**We performed a paired-sample t-test to compare the accuracies between the numerosity and luminance tasks, and found a significant difference (t(430) = -24.524, p < 0.001). This result suggested that the tasks were not equally difficult, with the numerosity task proving to be significantly more challenging, which raised the possibility of a confounding influence arising from task difficulty in the brain activation signal. Then, we calculated the difference in accuracy between the numerosity and luminance tasks for each participant, and conducted the Pearson correlation analyses between these accuracy differences and the PSC values across 15 ROIs. No significant correlations were observed in any of the examined brain regions (FDR corrected, all ps > 0.165). It thus lent support to the notion that task difficulty may not be the primary determinant of brain activation differences found in the present study.**

**Evaluation of functional probabilistic atlas**

We evaluated the reliability of our functional probabilistic atlas by examining four features of the atlas: anatomical correspondence, inter-rater reliability, sample size effect, similarity between meta-analytic maps and behavioral correlation.

***Anatomical correspondence***

The anatomical locations of the numerosity-related ROIs were characterized in relation to the macro-anatomical regions derived from the Harvard–Oxford cortical probabilistic atlases (Desikan et al. 2006), with a probability threshold of 0. For each subject, the spatial overlap between each ROI and each anatomical area was calculated as the number of voxels in their intersection divided by the number of voxels of that functional area. Then, this spatial overlap index was averaged across participants to characterize the correspondence between the numerosity-related ROIs and anatomical regions. Overlaps whose rates were less than 5% was considered to be unstable and therefore excluded from further analysis.

The averaged overlap rates of each pair of functional and anatomical regions were shown in a heat map (Figure S2). Our analysis revealed that each functional region was overlapped with at least two anatomical regions, indicating that there was no one-to-one correspondence between the functional regions and the Harvard–Oxford anatomical regions. For example, the right ITG overlapped with the middle temporal gyrus (MTG), the temporal inferior gyrus (tITG), and the inferior lateral occipital cortex (iLOC), with overlap rates of 29.6%, 35.0%, and 28.0%, respectively. Similarly, the right PM was overlapped with the superior frontal gyrus (SFG), middle frontal gyrus (MFG), and precentral gyrus (PreCG), with overlap rates of 31.6%, 50.0%, and 16.0%, respectively. These findings highlight the complex and distributed nature of the neural substrates involved in numerosity processing.

*
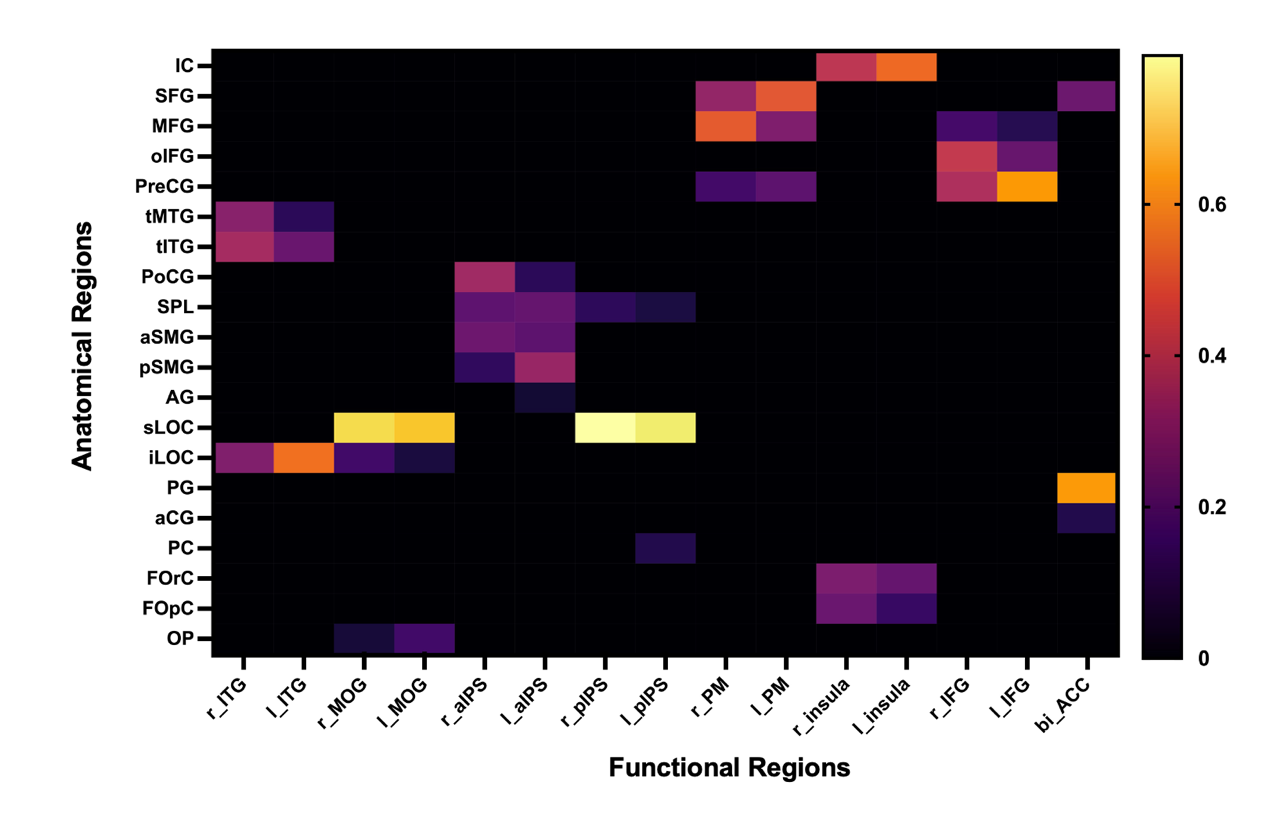
*

**Figure S2. Anatomical correspondence of the functional regions involved in numerosity processing.** The values presented in each cell of the heat map indicate the averaged overlap rate, which was calculated as the number of voxels in their intersection divided the number of voxels of corresponding functional region. Overlap rates below 5% were set to 0. IC, Insula Cortex; SFG, Superior Frontal Gyrus; MFG, Middle Frontal Gyrus; oIFG, Inferior Frontal Gyrus, pars opercularis; PreCG, Precentral Gyrus; tMTG, Middle Temporal Gyrus, temporooccipital part; tITG, Inferior Temporal Gyrus, temporooccipital part; PoCG, Postcentral Gyrus; SPL, Superior Parietal Lobule; aSMG, Supramarginal Gyrus, anterior division; pSMG, Supramarginal Gyrus, posterior division; AG, Angular Gyrus; sLOC, Lateral Occipital Cortex, superior division; iLOC, Lateral Occipital Cortex, inferior division; PG, Paracingulate Gyrus; aCG, Cingulate Gyrus, anterior division; PC, Precuneous Cortex; FOrC, Frontal Orbital Cortex; FopC, Frontal Operculum Cortex; OP, Occipital Pole.

***Inter-rater reliability and sample size effect***

To assess the inter-rater reliability of the ROI delineation procedure, we calculated Dice’s coefficient for each region between two raters. Dice’s coefficient was calculated using the following formula:

Dice = 2 ∗ (VA ∩ VB) ∕ (VA+VB)

where VA ∩ VB represents the number of overlapping voxels between the ROI results of rater A and B, and VA+VB indicates the sum of the number of voxels in these two ROI results. A value close to 1 indicates a marked overlap between two ROI results of the main and assistant raters, and 0 indicates no overlap. The results indicated that the Dice coefficients in all regions were close to or higher than 0.80, particularly in the ITG and ACC, suggesting that all ROIs had good inter-rater reliability and the two raters exhibited consistent performance in delineating these ROIs (Figure S3).


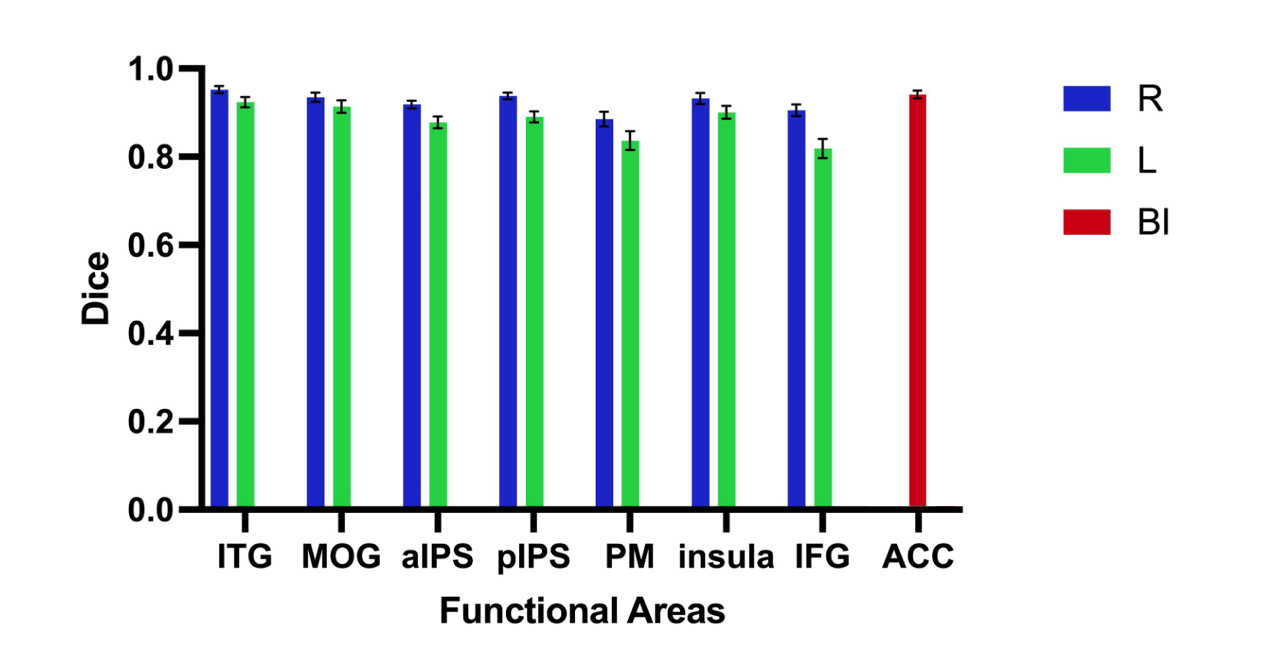


**Figure S3.** **Inter-rater reliability of subject-specific delineation measured by Dice’s coefficient across two raters.** Error bars indicate standard error of the mean.

Then, to evaluate the sample size effect, we created multiple functional probabilistic atlases using a reduced number of participants, ranging from 1 to 456. Dice’s coefficients were calculated to measure the overlap rates between the functional probabilistic atlas with reduced sample size and the atlas with all participants. The results showed that Dice’s coefficient was only around 60% when including 50 participants, but increased to nearly 100% when including 400 participants (Figure S4). These findings indicated that larger sample sizes have a strong effect on the stability of the atlas and suggested that previous fMRI studies that relied on small sample sizes may have produced imprecise estimates of numerosity-related activation regions. Therefore, we recommend using larger sample sizes to improve the reliability and accuracy of ROI delineation.


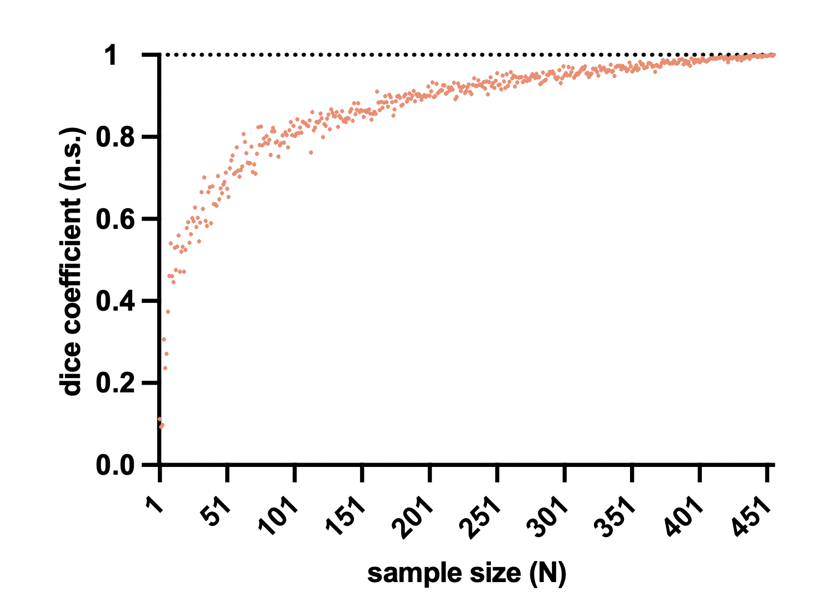


**Figure S4.** **Effect of sample size on the reliability of the probabilistic atlas.** Dice’s coefficient was calculated to measure the overlap between functional probabilistic atlas with all participants (N = 456) and atlas with reduced sample sizes (ranging from 1 to 456).

***Comparing functional probabilistic atlas with the uniformity meta-analytic map***

To evaluate the agreement between our functional probabilistic atlas and previous functional activation maps, we compared the boundaries of regions in our atlas with those of the uniformity meta-analytic reference map generated by topic-based analyses from Neurosynth (Yarkoni et al. 2011). Specifically, we selected the “v5-topic-400” set, which consists of 400 topics extracted with LDA (Latent Dirichlet Allocation) from abstracts of articles in the Neurosynth database as of July 2018, and identified one topic (topic 86) that contains the term “numerosity”. As the uniformity test map offers information on consistently active brain regions across studies, we downloaded this topic's uniformity test map to serve as the meta-analytic reference map for “numerosity” (Figure S5). The z-score of each voxel was calculated using a chi-square (χ^2^) test, where the null hypothesis assumes that activation in all the voxels was equally probable. Voxels with higher z-scores were more frequently reported in studies than other voxels.

We found that all regions acquired by meta-analyses overlapped with our functional probabilistic atlas, indicating its validity. However, it is worth noting that the activation volumes of numerosity-related regions in the map from Neurosynth were nearly the same in the left and right hemisphere, whereas our atlas showed significant hemispheric differences, suggesting that our probabilistic atlas may offer a more comprehensive and accurate depiction of the hemispheric specialization in numerosity processing.

*
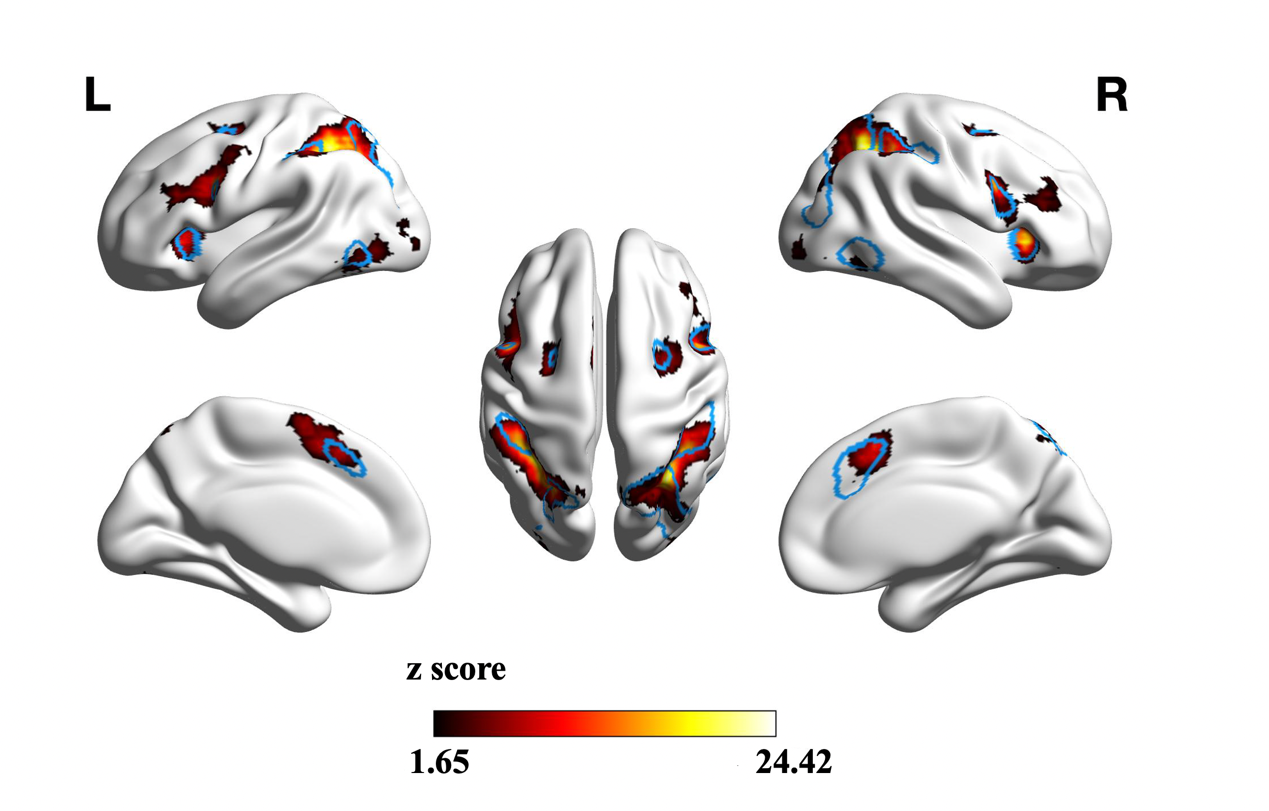
*

**Figure S5. Comparison of the meta-analytic functional map and functional probabilistic atlas.** The meta-analytic map was generated using Neurosynth. Values below 1.65 were set to zero. The blue contour shows the boundary of our functional probabilistic atlas to facilitate visual comparison. L, left hemisphere; R, right hemisphere.

**Sex differences in brain activation independent of GMV variations**

**Given the observed differences in GMV between males and females (Liu et al. 2020), the difference in the GMV between males and females might result in the difference in signal-to-noise ratio, which may also lead to different activation magnitude between males and females. We thus conducted a control analysis to exclude the potential confounding influence of GMV differences between males and females.**

**First, we processed the structural MRI data of all participants (N = 435, data from 21 participants were discarded due to image quality issue) using Voxel-Based Morphometry (VBM) in SPM8 (http://www.fil.ion.ucl.ac.uk/spm). Initially, T1-weighted anatomical images were segmented into gray matter (GM), white matter (WM), and cerebrospinal fluid using Ashburner and Friston's unified segmentation approach (Ashburner and Friston 2005). Subsequently, these segmented GM images were rigidly aligned and resampled to 2 x 2 x 2 mm. We then generated a study-specific GM template using the Exponential Lie Algebra (ELA) registration method (Ashburner 2007). Each GM image was normalized to this template within the Montreal Neurological Institute (MNI152) space. To preserve tissue volume, we modulated the GM values at each voxel by multiplying them with the Jacobian determinants obtained from the normalization procedure. Subsequently, these modulated GM images were smoothed using an 8-mm FWHM isotropic Gaussian kernel and subjected to an absolute threshold of 0.2 for masking. For the final analysis, these masked and modulated images were used. We computed the average gray matter volume (GMV) for each of the 15 ROIs created using the group-level functional reference map.**

**Second, we performed independent-samples t-tests to** **evaluate differences in GMV between males and females across each ROI. Our analysis revealed significant sex differences in all the ROIs examined (FDR-corrected, all p-values < 0.01), suggesting that sex differences in GMV could potentially contribute to the observed variations in brain activation, as measured by PSC.**

**Finally, to control for the potential confounding GMV differences between males and females, we followed the approach outlined by (DeGutis et al. 2013). Specifically, we conducted a regression analysis for each ROI, using the PSC within the ROI as the dependent variable and its corresponding GMV as the independent variable. The computed regression residuals thus capture the PSC-specific variation, having statistically controlled for GMV differences. Subsequently, we performed independent-samples t-tests on the regression residuals for each ROI to assess sex differences between males and females. The analysis revealed significant sex differences in 11 out of 15 ROIs, with males exhibiting notably stronger PSC compared to females (Fig. 8).**

**Reference:**

Ashburner J (2007) A fast diffeomorphic image registration algorithm. Neuroimage 38 (1):95-113. doi:10.1016/j.neuroimage.2007.07.007

Ashburner J, Friston KJ (2005) Unified segmentation. Neuroimage 26 (3):839-851. doi:10.1016/j.neuroimage.2005.02.018

DeGutis J, Wilmer J, Mercado RJ, Cohan S (2013) Using regression to measure holistic face processing reveals a strong link with face recognition ability. Cognition 126 (1):87-100. doi:10.1016/j.cognition.2012.09.004

Desikan RS, Ségonne F, Fischl B, Quinn BT, Dickerson BC, Blacker D, Buckner RL, Dale AM, Maguire RP, Hyman BT, Albert MS, Killiany RJ (2006) An automated labeling system for subdividing the human cerebral cortex on MRI scans into gyral based regions of interest. Neuroimage 31 (3):968-980. doi:10.1016/j.neuroimage.2006.01.021

Liu S, Seidlitz J, Blumenthal JD, Clasen LS, Raznahan A (2020) Integrative structural, functional, and transcriptomic analyses of sex-biased brain organization in humans. Proc Natl Acad Sci U S A 117 (31):18788-18798. doi:10.1073/pnas.1919091117

Yarkoni T, Poldrack RA, Nichols TE, Van Essen DC, Wager TD (2011) Large-scale automated synthesis of human functional neuroimaging data. Nat Methods 8 (8):665-670. doi:10.1038/nmeth.1635
